# Validation of the Italian Sense of Agency Scale and Its Relevance to Well-Being: An Iterative Bootstrap Approach

**DOI:** 10.3390/bs15010017

**Published:** 2024-12-28

**Authors:** Simone Di Plinio, Claudia Greco, Simone Arnò, Marco Innamorati, Sjoerd J. H. Ebisch

**Affiliations:** 1 Department of Neuroscience, Imaging and Clinical Sciences, “G. d’Annunzio” University of Chieti-Pescara, 66100 Chieti, Italy; claau_24@hotmail.it (C.G.); sjoerd.ebisch@unich.it (S.J.H.E.); 2 Institute of Advanced Biomedical Technologies, “G. d’Annunzio” University of Chieti-Pescara, 66100 Chieti, Italy; 3 Department of Human Sciences, Università Europea di Roma, 00163 Rome, Italy

**Keywords:** sense of agency, Italian adaptation, exploratory factor analysis, psychosis, identity, sense of self, hopelessness, convergent validity

## Abstract

The Sense of Agency (SoA) refers to the subjective experience of controlling one’s actions and the external events resulting from those actions. This study aimed to critically evaluate the construct validity, robustness, and clinical utility of the Sense of Agency Scale (SoAS) in an Italian-speaking population, using a data-driven approach to explore potential factor structures. A sample of 992 adults completed the SoAS alongside other validated psychological measures, allowing for a comprehensive analysis of the scale’s psychometric properties. An iterative process of exploratory and confirmatory factor analyses (EFA and CFA) was implemented to assess both two-factor and bifactor models. A refined 10-item version of the SoAS was established, comprising two distinct factors: Sense of Positive Agency (SoPA, five items) and Sense of Negative Agency (SoNA, five items). The results favored a two-factor solution supported by satisfying fit indices, factor loadings, and stability. The revised scale demonstrated strong internal consistency, with McDonald’s omega values of 0.79 (SoNA) and 0.87 (SoPA). This study extends the literature by providing evidence of the scale’s convergent and divergent validity, with significant correlations in the expected directions with constructs such as sense of self, hopelessness, impulsiveness, psychosis-like experiences, and schizotypal traits. These findings support the revised SoAS as a reliable and valid measure for assessing the Sense of Agency and highlight its broader applicability in understanding psychological and psychopathological constructs.

## 1. Introduction

The Sense of Agency (SoA) refers to the ability to control one’s actions and to be aware of the consequences arising from them (Dewey & Knoblich, 2014), including perceptions, emotions, thoughts, attitudes (Newen & Vogeley, 2003), and feelings of responsibility (Frith, 2014). Assessing SoA could be relevant for psychological diagnostic and therapeutic purposes as it is known to be positively related to health (Haggard, 2017) and negatively related to mental disorders, such as schizophrenia and depression (Moccia et al., 2023). Studies on the SoA began as early as the 1980s (Newman et al., 1983; Abramson et al., 1978) but, in recent years, the SoA construct has been revisited in relation to the study of consciousness. The SoA can be defined as “the awareness that I am the agent of my actions” (Synofzik et al., 2008, 2013), reflecting a subject’s general ability to distinguish actions performed by oneself from those performed by others. Initially, it was thought to be based solely on motor experiences, focusing on efferent copies of movements (Tsakiris et al., 2005). Subsequently, it has been portrayed as a process resulting from the integration of both motor and non-motor information, thus recognizing it as a multisensory phenomenon (Synofzik, 2015) solidly rooted in sensorimotor brain networks (Di Plinio et al., 2020).

There are direct and indirect measures of the SoA (De Houwer, 2003), referred to as “judgment” of agency, which is higher-level cognitive processing, and “feeling” of agency, resulting from lower-level motor processing, respectively (Haggard, 2017). However, the correlation between direct and indirect measures of agency remains to be clarified (Moore & Obhi, 2012; Imaizumi & Tanno, 2019). Direct measures include rating scales that explicitly ask about various aspects of SoA over a specific event (Dewey & Knoblich, 2014) and self-report questionnaires that address it as a trait (Tapal et al., 2017). By contrast, indirect evaluations concern implicit measures of the SoA; for instance, intentional binding (Haggard et al., 2002; Kong et al., 2024), which is a temporal illusion that the interval between an action and its effect is generally perceived to be shorter if the experienced agency over the action is higher compared to when it is lower (Moore & Obhi, 2012), or sensory attenuation (Hughes et al., 2013), which concerns a reduced intensity of the perception of self-initiated stimuli compared to those caused externally. Intentional binding effects can be observed, for example, by comparing voluntary to involuntary actions (Desantis et al., 2011) or actions with consistent consequences compared to actions with random consequences (Haggard et al., 2002; Tsakiris et al., 2005).

Currently, only a limited number of instruments are available to explicitly assess the SoA. One such tool is the Sense of Agency Rating Scale (Polito et al., 2013), which was designed to measure changes in general agency beliefs during hypnosis and, hence, is not suitable for evaluating SoA across different situations. Another tool is the subjective personal agency scale (Yamaguchi et al., 2020), developed to measure SoA in individuals with severe mental disorders, particularly schizophrenia. To measure the SoA in the general population, Tapal et al. (Tapal et al., 2017) proposed more recently the Sense of Agency Scale (SoAS). The development of the SoAS started from an initial set of 36 items, 11 of which were selected on a content validity criterion. The final 11-item version of the scale was validated by comparing it with other constructs such as locus of control (Rotter, 1966), self-efficacy (Bandura, 1977), and sense of control (Lachman & Weaver, 1998).

Tapal and colleagues identified two opposite factors of agency and not a general factor. The Sense of Positive Agency (SoPA) factor includes items that refer to the level of control over one’s mind, feelings, body, and the environment, reflecting personal autonomy and responsibility over one’s own actions. The Sense of Negative Agency (SoNA) factors instead mention the lack of control over the same elements, reflecting the feeling of existential helplessness. SoPA and SoNA were only moderately correlated (Pearson r = 0.30), suggesting that they represent two distinct factors that measure unique components of a “global”, continuous SoA. Reliability was estimated at (McDonald’s) ω = 0.80 (95% CI = 0.76, 0.83) and ω = 0.75 (95% CI = 0.67, 0.79) for the SoPA and SoNA, respectively.

To date, few studies have investigated the psychometric properties, clinically relevant associations, and cultural adaptations of the SoAS. A French translation (Hurault et al., 2020) proposed a reduced seven-item scale since other items had weak factor loadings. The analyses confirmed a two-factor structure with limited reliability (McDonald’s ω = 0.65 for SoPA and ω = 0.53 for SoNA). A German adaptation and validation of a reduced 10-item version of the SoAS also indicated that some items were to be removed because of weak factor loadings on the proposed dimensions (Bart et al., 2023). They performed a confirmatory factor analysis on a one-factor model that resulted in a poor fit to the data. Contrasting the French study, the two-factor structure showed sufficient to good reliability (McDonald’s) of ω = 0.72 for SoPA and 0.85 for SoNA. Both the French and German studies found a gender difference, with lower scores in SoPA and higher in SoNA for females compared to males. Both translations report poor construct validity, together with inconsistency in the results with some items. Additionally, past studies did not test alternative bifactor or single-factor structures.

In this study, we aimed to validate an Italian version of the Sense of Agency Scale (SoAS), addressing the psychometric inconsistencies and limitations identified in previous research. Our study aimed to enhance the reliability, construct validity, and clinical utility of the SoAS by employing advanced psychometric analyses. These included exploratory and confirmatory factor analyses (EFA and CFA) and the evaluation of both two-factor and bifactor models to explore potential hierarchical structures of the scale. This methodological approach aligns with contemporary psychometric theories and ensures a robust, data-driven assessment of the SoAS’s validity.

Based on previous research, we anticipated identifying a stable two-factor structure, with the possible presence of a general factor. Additionally, we hypothesized that our iterative bootstrap approach, combined with EFA and CFA, would provide a more sensitive identification of problematic items, offering a rigorous and unbiased assessment of the scale’s psychometric properties. This approach ensures that model selection is both theoretically grounded and data-driven, providing a comprehensive evaluation of the scale’s structure. By using this method, we can confidently identify the most appropriate model while addressing issues such as cross-loadings and ensuring a robust fit to the data. Finally, we expected strong correlations between the SoPA and SoNA factors and measures of the sense of self, as well as traits with significant psychopathological relevance in the context of psychosis, depression, and impulsivity, particularly for SoNA. This comprehensive approach not only reinforces the construct validity of the SoAS but also ensures that the psychometric properties of the scale are tested rigorously and systematically. By utilizing an iterative model-testing procedure, we aim to provide a more robust tool for both research and practical applications in diverse psychological contexts, with a strong emphasis on reliability and clinical utility

## 2. Materials and Methods

### 2.1. Participants

This study collected data from 992 Italian participants ranging in age from 18 to 69 years (197 males, 781 females, 14 non-binaries), recruited from various areas across Italy through both social networks and physical advertising (scannable QR codes) posted in university campuses and public places. Inclusion criteria for all participants were age above 18, Italian as a native language, and consent to participate in the study. Exclusion criteria included any neurological disorder affecting cognitive functioning and any conditions affecting the ability to complete the assessment, including denial of informed consent. This study was approved by the local Ethical Committee of the University G. d’Annunzio of Chieti-Pescara, and all procedures were carried out in accordance with the Declaration of Helsinki. Participants were assured of their right to withdraw from the study at any time without any consequences and were informed about the study’s objectives and the confidential nature of their responses through the online platform Qualtrics. After providing written consent, participants completed the Italian version of the SoAS (Tapal et al., 2017) along with additional psychological scales to examine convergent and divergent validity. Demographic information is shown in Table 1.

### 2.2. Measures

All the participants were administered the Italian version of the Sense of Agency Scale. Subsets of the participants also completed the Beck Hopelessness Scale (BHS, Beck et al., 1974) (N = 691; Cronbach α = 0.91, M = 7.66 ± 5.51), the Barratt Impulsiveness Scale (BIS, Barratt, 1959, 1965) (N = 485; Cronbach α = 0.80, M = 60.6 ± 10.3), the Empathic Experience Scale, (EES, Innamorati et al., 2019) (N = 480; subscale Vicarious Experience: Cronbach α = 0.90, M = 40.2 ± 10.5; subscale Intuitive Understanding: Cronbach α = 0.94, M = 54.1 ± 9.87), the Empathy Quotient scale (EQ, Baron-Cohen & Wheelwright, 2004) (N = 161; Cronbach α = 0.63, M = 32.8 ± 5.61), the Community Assessment of Psychic Experience (CAPE, Mossaheb et al., 2012) (N = 983; subscale Positive: Cronbach α = 0.85, M = 40.0 ± 7.01; subscale Depressive: Cronbach α = 0.87, M = 19.2 ± 4.78; subscale Negative: Cronbach α = 0.89, M = 29.6 ± 7.69), the Aspects of Identity Questionnaire (AIQ, Cheek & Briggs, 1982; Cheek & Cheek, 2018) (N = 228; subscale Personal Identity: Cronbach α = 0.83, M = 40.4 ± 6.21; subscale Relational Identity: Cronbach α = 0.90, M = 38.7 ± 7.33; subscale Social Identity: Cronbach α = 0.78, M = 19.1 ± 5.00; subscale Collective Identity: Cronbach α = 0.75, M = 20.1 ± 5.93), the Schizotypal Personality Questionnaire (SPQ, Raine, 1991) (N = 439; subscale Cognitive-Perceptual: Cronbach α = 0.73, M = 2.40 ± 2.11; subscale Interpersonal: Cronbach α = 0.72, M = 3.98 ± 2.23; subscale Disorganized: Cronbach α = 0.67, M = 2.17 ± 1.75), and the Sense of Self Inventory (SoSI, Di Plinio et al., 2024) (N = 279; subscale Self-Trait: Cronbach α = 0.84, M = 41.1 ± 12.3; subscale State-Identity: Cronbach α = 0.85, M = 39.1 ± 12.0; subscale State-Agency: Cronbach α = 0.83, M = 34.8 ± 9.3; subscale State-Technology: Cronbach α = 0.76, M = 22.0 ± 6.88).

The SoAS was translated into Italian by bilingual academics with a background in psychology. The translation process followed a standard back-translation method to ensure the accuracy of the psychological terminology and the conceptual equivalence between the English and Italian versions.

#### 2.2.1. Iterative Exploratory and Confirmatory Factor Analyses

Data analysis was performed in MATLAB (Version R2022b), R (Rstudio Version 2024.04.0, build 735; packages lavaan, psych, R.matlab), and JASP (0.19.1). Prior to data analysis, outliers were identified and excluded from the dataset. Listwise deletions were used for missing data..

To investigate the dimensionality of the SoAS, we used an iterative exploratory/confirmatory factorial approach. We generated 1000 bootstrap samples by randomly splitting the original dataset into two halves for each iteration. For each iteration, we performed an exploratory factor analysis (EFA) on the first half of the data and conducted a confirmatory factor analysis (CFA) on the second half of the data to confirm the factor structure identified in the EFA. Results from all the iterations were analyzed to assess the stability and robustness of the psychometric properties of the scale. We restricted the analyses to a two-factor solution, in line with theoretical expectations. A minimum loading threshold of 0.40 was used to retain significant EFA loadings, and cross-loading items were removed (cross-loading >= 0.30). For the CFA, cross-loading items and below-threshold loadings were set to zero, and items with loadings above the threshold were retained and used to define the two underlying factors. A maximum likelihood robust estimator (MLR) on a correlation matrix was used for the analyses.

We chose to use an iterative non-parametric bootstrapping without replacement procedure for this exploratory factor analysis to ensure rigorous testing of the factor structure robustness, providing a reliable foundation for further confirmatory factor analysis and theoretical investigations into the SoA constructs (Costello & Osborne, 2005).

At each cycle, the Chi-squared statistic, Comparative Fit Index (CFI), and root mean square error of approximation (RMSEA) were used to assess the goodness-of-fit of the two-factor model. The distribution of both coefficient-rho and Cronbach’s alpha values across the iterative process were calculated and examined, providing insights into the reliability of the factors. Additionally, unique factor combinations and their frequency were analyzed and displayed to highlight the most stable item groupings within each factor. This method enhances the reliability of the factor loadings by ensuring that the factors extracted are consistent across different segments of the total sample. This is crucial because it helps to identify and confirm the underlying structure of the scale without biases and provides a strong foundation for the subsequent SEM (Kline, 2015).

#### 2.2.2. Structural Equation Modeling

Structural Equation Modeling (SEM) analysis on the whole sample was performed to compare the fit of the models suggested through the iterative exploratory and confirmatory factor analysis illustrated above. The selection of the final model was based on fitting the data, parsimony considerations, and theoretical justification of the model structure.

#### 2.2.3. Validity Assessment

Once we assessed the final structure of the Italian version of the SoAS, the scale’s stability and criterion validity were assessed using Pearson correlation coefficients between SoPA and SoNA subscales and other psychological measures. To test whether SoNA and SoPA dimensions exhibited discriminant validity, we utilized an ad hoc-developed procedure that integrates bootstrap and effect size estimation (Di Plinio, 2022). This method involves calculating the bootstrap confidence intervals for the differences between correlation coefficients to determine if the differences are statistically significant. The statistical analyses were conducted using MATLAB, with the bootstrap procedures implemented via the bootes function available from MathWorks. Uncorrected significance thresholds were hierarchically set at *p* < 0.001, *p* < 0.01, and *p* < 0.05.

## 3. Results

### 3.1. Results from Iterative Exploratory and Confirmatory Factor Analyses

**Figure 1:**
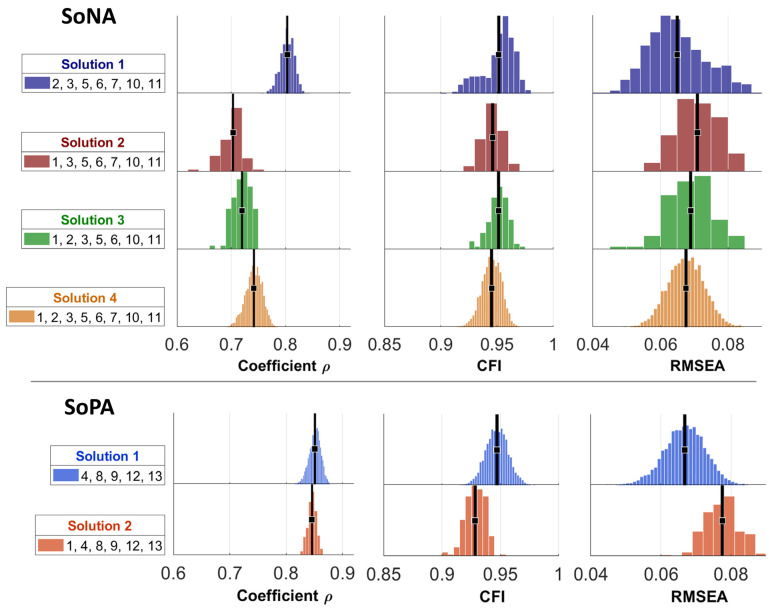
Results of the iterative exploratory and confirmatory factor analyses (EFA, CFA). The distributions represent reliability (rho) for EFA and confirmative fit index (CFI) and root mean square of the error of approximation (RMSEA) for CFA across different factor solutions. Each color represents a different solution: Solution 1 (blue), Solution 2 (red), Solution 3 (green), and Solution 4 (orange) for SoNA; Solution 1 (light blue) and Solution 2 (light red) for SoPA. The x-axis shows the range of the respective metrics, while the y-axis shows the frequency within bootstrap samples. Black vertical lines indicate the mean values for each distribution.

For factor 1, related to the original factor Sense of Negative Agency (SoNA), we observed high levels of reliability across simulations. However, this factor unexpectedly included item 1, which was originally anticipated to align with the Sense of Positive Agency (SoPA). Additionally, the inclusion of items 1, 2, and 7 was found to be unstable. The root mean square error of approximation (RMSEA) from confirmatory factor analysis (CFA) showed varying degrees of error across different solutions. The Comparative Fit Index (CFI) suggested a higher fit when the unstable items (1, 2, and 7) were excluded from the analyses. For factor 2, items from the SoPA (items 4, 8, 9, 12, and 13) were consistently included, but item 1 was excluded in 96% of the iterations. The inclusion of item 1 occurred only in 4% of the samples, indicating its instability. Indeed, the CFA (RMSEA) indicated a better solution when item 1 was excluded from the SoPA. Correspondingly, the CFI highlighted an improved fit with only items 4, 8, 9, 12, and 13. The solutions for the two factors are reported in Figure 1, along with relevant statistics across the 500 cycles. Importantly, crossloadings (>0.30) were also inspected in each of the 5000 cycles, and it was found that the error term of item 1 crossloaded with other items in 52% of the cases. Any other item’s error term crossloaded in less than 5% of the cycles. For these reasons, we excluded item 1 from further consideration.

Given the results from the iterative EFA and CFA, we tested and compared the following five models on the whole sample: (1) model M12 included all the original SoAS items except item 1 and consisted of a SoNA scale (items 2, 3, 5, 6, 7, 10, and 11) and a SoPA scale (items 4, 8, 9, 12, and 13); (2) model M11_no2, which additionally excluded item 2; (3) model M11_no7, which excluded item 7; (4) model M10, consisting of 10 items and excluding both items 2 and 7; and finally, (5) model M10b, which consisted of the same 10 items but included a bifactorial structure with uncorrelated factors. The bifactor model (M10b) was included to explore whether a general Sense of Agency could be captured as a latent trait influencing all items, alongside specific factors for Positive and Negative Agency. This model allowed us to assess the hierarchical nature of the scale and the potential contributions of a general factor alongside domain-specific factors. These models were evaluated and compared to determine the most stable and fitting structure for the SoAS.

### 3.2. Results from Structural Equation Modeling

Table 2 lists the summary of relevant fit indices for the models tested. Although all models demonstrated a good fit, models M10 and M10b showed the highest CFI, TLI, and Normed Fit Index (NFI), with values above 0.95. Specifically, M10 also scored well on the Parsimony Fit Index, in contrast to model M10b. The bifactor structure in M10b effectively captured the variance and structure of the data, potentially indicating a general latent trait affecting all questionnaire items along with specific factors for unique aspects. Both M10 and M10b exhibited the highest quality of fit based on various indices, including the RMSEA, the Expected Cross-Validation Index (ECVI), McDonald’s Fit Index (MFI), and Hoelter’s Critical N. These indices indicated strong adherence to the current data and good predictive power.

Looking at the general fit indices for the models (Table 3), models M10 and M10b showed the lowest values for the Akaike Information Criterion (AIC) and the Bayesian Information Criterion (BIC), indicating an optimal balance between fit and complexity. In contrast, models M11_no2, M11_no7, and M12 had significantly higher AIC and BIC values, suggesting that fit to the data was obtained at the expense of greater complexity or less efficiency. All models showed a significant Chi-squared test, indicating significant deviations from a perfect fit, which is a common outcome in large samples where even trivial discrepancies can result in significant *p*-values (Alavi et al., 2020; Bergh, 2015). While the bifactor model, M10b, demonstrated strong fit indices, including high CFI, TLI, and RMSEA values, it was ultimately rejected due to problematic factor loadings. Specifically, although the loadings on the general factor and the SoPA factor were high (loadings > 0.4), the SoNA factor in the bifactor model showed low (<0.20) or sign-reversed loadings, undermining the model’s interpretability. Consequently, the overall results suggest that model M10, with consistently high fit indices and more interpretable loadings, provides the most efficient structure for the SoAS.

Overall, we chose model M10 as the best model since it showed the highest fit indices (CFI, TLI, NFI), all above 0.95, and excelled particularly with a strong Parsimony Fit Index, unlike M10b. Model M10 exhibited good performance across other fit indices (RMSEA, ECVI, MFI, and Hoelter’s Critical N), indicating robust adherence to the data and strong predictive capability. Although model M10b demonstrated an effective bifactor structure and strong fit indices, it was ultimately hampered by low or sign-reversed loadings on the Sense of Negative Agency (SoNA) factor. These problematic loadings made the bifactor model less interpretable and unsuitable for representing the structure of the scale. In contrast, model M10 had consistently strong loadings across all items and a simpler, more interpretable structure, making it the most appropriate choice. The items of this Italian version of the SoAS and their loadings are reported in Table 4. Both underlying factors showed high reliability, with a McDonald’s ω of 0.798 for the Sense of Negative Agency and 0.866 for the Sense of Positive Agency. More information can be found in Table 5, which compares the reliability of the two factors across different versions of the SoAS.

### 3.3. Construct Validity Results

Correlations between the SoAS and other questionnaires are shown in Table 6 and generally show a high correlation of SoAS with questionnaires describing subclinical symptoms or other aspects of the sense of self, confirming the convergent validity of the SoAS as a good psychometric instrument. Despite the two factors showing a similar pattern of correlations, some differences are worth being discussed. The first factor resulting from our analyses, Sense of Negative Agency (SoNA), included almost all items in the negative form and showed higher correlations with impulsiveness (BIS), frequency of psychic experiences (CAPE), schizotypal traits (SPQ), and sense of self (SoSI). In turn, the second factor, Sense of Positive Agency (SoPA), included items in the positive form and showed higher correlations with the Personal Identity subscale of the Aspects of Identity Questionnaire (AIQ). Moreover, both subscales showed low correlations with empathy (EES) and socioemotional skills (EQ).

## 4. Discussion and Conclusions

The Sense of Agency (SoA), defined as the capacity to recognize and control one’s actions and their consequences (Dewey & Knoblich, 2014; Moore, 2016), plays a pivotal role in psychological well-being. Disruptions in SoA have been linked to the presence of psychotic and depressive symptoms (Keeton et al., 2008; Asai & Tanno, 2007) and even to broader psychopathological conditions (Moccia et al., 2023). This underscores the critical need for valid and reliable tools to measure SoA, not only within clinical populations but also in the general population. However, existing psychometric instruments for evaluating individual differences in SoA are limited and exhibit considerable variability across different cultural adaptations. The original Sense of Agency Scale (SoAS) by Tapal et al. (Tapal et al., 2017) introduced an 11-item measure, which has since undergone various modifications in different languages—such as the 10-item German adaptation (Bart et al., 2023) and the seven-item French version (Hurault et al., 2020)—each retaining the two-factor structure of Sense of Positive Agency (SoPA) and Sense of Negative Agency (SoNA).

A significant limitation of these adaptations is the inconsistency in item selection, suggesting potential instability in the measurement of SoA across different cultural contexts. Moreover, prior studies did not fully assess the stability of the factor structure, particularly with respect to hierarchical or bifactorial models, leaving gaps in our understanding of the scale’s construct validity. In addressing these issues, this present study translated and validated the Italian version of the SoAS and conducted a thorough investigation of various factor models to identify the most robust structure that best fits the data. By doing so, our research informs upon the scale’s construct validity, provides a more robust solution, and enhances its clinical utility. Specifically, the validation process confirmed the stability and reliability of a refined 10-item version of the SoAS, reinforcing its applicability for assessing SoA in both clinical and non-clinical populations and providing a more robust tool for future research and clinical assessments.

To address the potential instability of SoAS items, we used iterative non-parametric bootstrapping and explored the solution space, enabling a robust estimation of factor loadings, reliability, and fit indices. In contrast, traditional split-half methods typically involve a single arbitrary or random division of the sample, which can introduce bias and variability depending on the specific split used (Floyd & Widaman, 1995). Our iterative bootstrap approach overcomes these issues by generating multiple samples of EFA–CFA combinations, ensuring a comprehensive evaluation of the factor structure (Ren et al., 2010; Gerbing & Hamilton, 1996). Aggregating the results from all iterations provides a detailed analysis of the psychometric properties across different subsamples and strengthens the foundation for subsequent Structural Equation Modeling (Marsh et al., 2014). Subsequently, applying SEM to the entire sample leverages the full dataset and enhances the statistical power and generalizability of our results. This method allows for sophisticated model comparisons and a more accurate confirmation of the theoretical models suggested by the iterative EFA and CFA phases (Schmitt, 2011).

In line with previous studies (Tapal et al., 2017; Hurault et al., 2020; Bart et al., 2023), we confirmed a two-factor model with a Positive Agency factor (Sense of Positive Agency, SoPA) and a Negative Agency factor (Sense of Negative Agency, SoNA). Furthermore, we demonstrated how model fit changes with the inclusion or exclusion of some problematic items. Our findings are in line with previous studies of the SoAS, so that items categorized under the first factor (SoNA) highlight aspects associated with a perceived lack of control, agency, or personal efficacy, where actions are not fully intentional or are swayed by external influences. On the other hand, the items under the second factor (SoPA) focus on aspects of personal control and responsibility for one’s actions, indicating a dimension of the scale that captures an active and intentional experience of agency. One exception regarding SoNA is about the observation of satisfying levels of reliability in those cycles in which item 1 (“I am in full control of what I do”), originally considered a “Positive” item, was included in the SoNA. We speculate that this change may indicate a cultural difference in how this item is perceived or understood within the Italian cultural context compared to the original setting, or that it may reflect instability of the original items. In addition, we observed that item 7 (“The outcomes of my actions generally surprise me”) had unstable SoNA factor loadings and would not be always beneficial for the factor reliability, which is coherent with the German and French analyses and validations. Also, item 2 (“I am just an instrument in the hands of somebody or something else”) was characterized by doubtful SoNA factor loadings.

In our analysis, we used confirmatory factor analysis with Structural Equation Modeling (SEM) to compare five different models, including two-factor and bifactor structures. These models were tested both with and without the inclusion of items that were identified as potentially crucial for model stability. The best-performing models were found to be the two-factor and bifactor models excluding items 1, 2, and 7, as evidenced by high values on the Comparative Fit Index (CFI), Tucker–Lewis Index (TLI), and Normed Fit Index (NFI), all exceeding 0.95. While the bifactor model demonstrated good overall fit indices, its loadings for the Sense of Negative Agency (SoNA) factor were problematic, with some loadings being low or even sign-reversed. This made the bifactor model less interpretable and raised concerns about its ability to accurately capture the structure of the SoAS. In contrast, the two-factor model demonstrated strong performance not only on the fit indices but also in terms of its interpretability, particularly with a robust Parsimony Fit Index and consistently strong loadings for both factors. Given these considerations, we selected the two-factor model excluding items 1, 2, and 7 as the most appropriate fit for the scale. This choice reflects the distinct and complementary roles of the SoPA and SoNA factors in capturing the nuances of the Sense of Agency. The exclusion of specific items further highlights the need to refine and adapt the scale for the Italian version of the SoAS. Our findings underscore the importance of balancing model fit with interpretability, emphasizing the value of precise model selection and item inclusion to ensure robust measurement tools.

Regarding the validation of the SoAS, we found strong correlations between the subscales SoPA and SoNA with measures of hopelessness and schizotypal traits (assessed by BIS, CAPE, and SPQ), confirming the convergent validity of SoAS. Strong correlations with general measures of the Sense of Self Inventory (SoSI) further suggest that the SoA is an integral part of ipseity.

When comparing the two subscales, we observed distinct patterns. SoNA exhibited higher positive correlations with scales measuring impulsiveness (BIS), frequency of psychosis-like experiences (CAPE), schizotypal traits (SPQ), and higher negative correlations with the Sense of Self Inventory (SoSI). Conversely, SoPA showed stronger correlations with the “personal identity” subscale of the Aspect of Identity Questionnaire (AIQ), which assesses personal features, traits, and values, along with one’s internal perception of identity. These results suggest that SoNA is particularly associated with measures related to the sense of self and neuropsychological integrity, whereas SoPA is more involved in motivation and self-esteem. Overall, these findings confirm the validity of the agency measures of SoAS and underscore the importance of an intact SoA for the construction of identity and sense of self. Both SoNA and SoPA showed low correlations with empathy (EES) and socioemotional skills (EQ), indicating that SoA is not linked to the ability to feel and understand others’ emotions. This aligns with the observations made by Tapal and colleagues when they first developed the scale.

Despite the significant findings and contributions of this study, several limitations must be acknowledged. First, the sample was predominantly female, which may affect the generalizability of the results to the broader Italian population. Second, the cross-sectional nature of this study limits the ability to draw causal inferences between the Sense of Agency and associated psychological constructs. Longitudinal studies would be beneficial to examine the temporal stability of the SoAS and the directionality of its relationship with other variables. Third, although the psychometric properties of the SoAS were rigorously evaluated, this study did not include follow-up data to assess test–retest reliability. Future research incorporating longitudinal designs would provide valuable insights into the scale’s stability over time. Fourth, the criterion validity of the SoAS was not explored in this study, as comparisons between contrasting groups were beyond its scope. Future investigations should aim to assess the discriminative capacity of the scale across diverse populations and contexts. Finally, together with past validation studies, we highlighted cultural differences in the item composition of the SoPA and SoNA scales, indicating the necessity for careful consideration in cross-cultural adaptations and validations of the SoAS.

Overall, we provide a reliable exploration of the SoAS, ensuring a robust and theoretically sound factor structure in a large sample and providing a solid foundation for further theoretical investigations and practical applications. The SoA has distinct components that can influence various aspects of personality and psychological experiences with potential clinical relevance, including major psychiatric disorders. Future psychometric studies are needed to confirm consistency within as well as across different cultural contexts. The distinction between a negative and a positive SoA is crucial for understanding how different individuals perceive and react to their sense of control and the intentionality of their actions. Particularly, SoNA highlights how a sense of loss of control can be associated with potential problematic or psychopathological traits, such as increased impulsiveness, misalignment with perceived reality, paranoia and magical thinking, depressive symptoms, and negative aspects of Personal Identity. On the other hand, SoPA emphasizes the importance of a positive SoA for a healthy and integrated perception of Personal Identity. It is important then to consider both the positive and negative dimensions of agency to evaluate individual strengths and vulnerabilities in the general population.

## Figures and Tables

**Table 1:** Sociodemographic features of the sample.

N = 992 Italian Participants	
AGE	
Mean	27.1
SD	8.4
Min, Max	18, 69
GENDER	
Male	197 (19.9%)
Female	781 (78.7%)
Non Binary	14 (1.4%)
OCCUPATION	
Student	543 (54.7%)
Working student	80 (8.1%)
Stable job	224 (22.6%)
Temporary job	92 (9.3%)
Unemployed	53 (5.3%)
EDUCATION	
Middle School	39 (3.9%)
High School	434 (43.8%)
Bachelor’s Degree	323 (32.6%)
Master’s Degree	147 (14.8%)
Postgraduate	49 (4.9%)

**Table 2:** Fit indices across the three models tested using SEM.

Index	M12	M11_no2	M11_no7	M10	M10b
Comparative Fit Index (CFI)	0.962	0.965	0.97	0.974	0.988
T-sized CFI	0.946	0.949	0.956	0.96	0.977
Tucker–Lewis Index (TLI)	0.952	0.956	0.962	0.966	0.978
Normed Fit Index (NFI)	0.951	0.956	0.961	0.966	0.982
RMSEA	0.059	0.059	0.056	0.056	0.045
RMSEA *p*-value	0.032	0.036	0.12	0.15	0.728
SRMR	0.039	0.038	0.036	0.034	0.021
Hoelter’s Critical N (α = 0.05)	303	306	334	347	493
Goodness-of-fit Index (GFI)	0.962	0.965	0.969	0.972	0.985
McDonald Fit Index (MFI)	0.913	0.927	0.935	0.948	0.975
Expected CV Index (ECVI)	0.286	0.241	0.224	0.183	0.137
Parsimony NFI (PNFI)	0.764	0.747	0.751	0.73	0.545

**Table 3:** Model general fit measurements, baseline tests, and difference tests.

				Baseline Test			Difference Test		
Model	AIC	BIC	N	Χ^2^	df	*p*	Χ^2^	df	*p*
M10b	30,678	30,825	992	233.2	53	<0.001	-	-	-
M10	30,724	30,827	992	139.2	34	<0.001	63.2	9	<0.001
M11_no2	34,037	34,150	992	76.0	25	<0.001	53.4	9	<0.001
M11_no7	33,680	33,793	992	192.6	43	<0.001	−16.1	0	-
M12	36,993	37,116	992	176.5	43	<0.001	56.7	10	<0.001

**Table 4:** Loading and Cronbach’s reliability (if item dropped) of the final Italian SoAS.

N	Item		Loading		α If Dropped	
	English	Italian	SoNA	SoPA	SoNA	SoPA
3	*My actions just happen without my intention*	*Svolgo le mie azioni senza una mia reale intenzionalità*	0.726	-	0.735 (−0.058)	-
4	*I am the author of my actions*	*Io sono l’artefice delle mie azioni*	-	0.727	-	0.842 (−0.019)
5	*The consequences of my actions feel like they don’t logically follow my actions*	*Le conseguenze delle mie azioni sembrano non seguire in maniera logica le mie stesse azioni*	0.599	-	0.768 (−0.025)	-
6	*My movements are automatic—my body simply makes them*	*I miei movimenti sono automatici; il mio corpo li esegue semplicemente*	0.582	-	0.771 (−0.022)	-
8	*Things I do are subject only to my free will*	*Le cose che faccio dipendono solo dal mio libero arbitrio*	-	0.740	-	0.832 (−0.029)
9	*The decision whether and when to act is within my hands*	*La decisione su se e quando agire è nelle mie mani*	-	0.846	-	0.811 (−0.050)
10	*Nothing I do is actually voluntary*	*Nulla di tutto ciò che faccio è veramente volontario*	0.741	-	0.746 (−0.047)	-
11	*While I am in action, I feel like I am a remote controlled robot*	*Mi sento come se fossi un robot radiocomandato durante lo svolgimento delle mie azioni*	0.677	-	0.750 (−0.043)	-
12	*My behavior is planned by me from the very beginning to the very end*	*Il mio comportamento è pianificato da me, dall’inizio alla fine*	-	0.800	-	0.817 (−0.044)
13	*I am completely responsible for everything that results from my actions*	*Sono pienamente responsabile di tutte le conseguenze delle mie azioni*	-	0.629	-	0.854 (−0.007)

**Table 5:** Reliability (McDonald’s ω) of the final Italian SoAS compared to the other versions.

	Italian	Ebrew	French	German
SoNA	0.793 [0.772 0.813]	0.75	0.53	0.83
SoPA	0.866 [0.852 0.879]	0.80	0.65	0.72

**Table 6:** SoAS constructs correlations with other relevant scales and differences in correlation strengths (Cohen’s *d*) between SoPA and SoNA using bootstrapped effect size. The asterisks in the SoNA and SoPA columns mark the significance of correlation with the other scales (*: *p* < 0.05; **: *p* < 0.01; ***: *p* < 0.001). The asterisks in the *d* column mark significant differences between SoPA and SoNA correlations with other factors (see Di Plinio, 2022).

Scale	SoNA	SoPA	*d*	n
BHS	0.37 ***	−0.21 ***	4.5 *	691
BIS	0.54 ***	−0.36 ***	4.9 *	485
CAPE: Positive	0.52 ***	−0.29 ***	8.1 *	983
CAPE: Depressive	0.48 ***	−0.36 ***	3.9 *	983
CAPE: Negative	0.57 ***	−0.40 ***	6.3 *	983
EES: Vicarious Experience	0.12 **	−0.05	1.5	480
EES: Intuitive Understanding	−0.09	0.09	0.1	480
EQ: Cognitive Empathy	−0.15	0.17 *	0.2	161
EQ: Emotional Reactivity	−0.11	0.05	0.8	144
EQ: Social Skills	0.09	−0.08	0.2	145
AIQ: Personal Identity	−0.23 ***	0.38 ***	2.5 *	228
AIQ: Relational Identity	−0.21 **	0.25 ***	0.5	228
AIQ: Social Identity	−0.11	0.12	0.04	228
AIQ: Collective Identity	0.01	0.09	1.4	228
SPQ: Cognitive	0.49 ***	−0.26 ***	5.3 *	439
SPQ: Interpersonal	0.41 ***	−0.26 ***	3.5 *	439
SPQ: Disorganized	0.48 ***	−0.25 ***	5.5 *	439
SoSI: Trait Self	−0.51 ***	0.43 ***	1.7	279
SoSI: State Identity	−0.62 ***	0.42 ***	4.4 *	279
SoSI: State Agency	−0.57 ***	0.44 ***	2.6 *	279
SoSI: State Technology	−0.46 ***	0.31 ***	3.1 *	279

## Data Availability

Data and code used for the study are available upon direct and reasonable requests to the authors.
